# Smoking history and the rate of hearing decline in aging: Results from a longitudinal cohort study

**DOI:** 10.1016/j.heares.2025.109486

**Published:** 2025-11-24

**Authors:** Lauren K Dillard, Lois J Matthews, Kathleen E Bainbridge, Jada M Johnson, Judy R Dubno

**Affiliations:** a Department of Otolaryngology- Head & Neck Surgery, Medical University of South Carolina, Charleston, SC, USA; b National Institute on Deafness and Other Communication Disorders, NIH, Bethesda, MD, USA

**Keywords:** Hearing loss, Smoking, Cohort study, Epidemiology, Aging, Prevention

## Abstract

Hearing loss is a common and impactful condition among aging adults. Improved understanding of modifiable risk factors for hearing loss, including smoking, could promote hearing loss prevention. The purpose of this longitudinal study was to determine the association of smoking with the rate of age-related hearing decline across the frequency range. Participants were from the Medical University of South Carolina Longitudinal Cohort Study of Age-related Hearing Loss. Smoking pack years were calculated (at baseline) as the number of self-reported packs smoked per day multiplied by years smoked, and categorized as 0, >0 to 5, >5 to 15, and >15 pack years. Outcome measures were individual audiometric thresholds (0.25 to 8.0 kHz) and pure-tone average (PTA) of thresholds at frequencies 0.5 to −4.0 kHz, averaged bilaterally. We used linear mixed regression models to determine the association of pack years with the rate of annual threshold change at each frequency and PTA. This study included 1032 participants (mean age: 63.3 [SD 14.1] years, mean follow-up time: 5.4 [SD 6.0] years; 60.4 % female; 21.7 % Black race). Compared to non-smokers (0 pack years), participants with >15 pack years had poorer baseline thresholds at frequencies 2.0 to 8.0 kHz and PTA. Compared to non-smokers, participants with >5 to 15 and >15 pack years had higher rates of annual threshold change at frequencies ranging from 1.0 to 8.0 kHz and PTA. Findings corroborate smoking as a possible modifiable risk factor for hearing loss that could have lasting impacts on hearing.

## 1. Introduction

Hearing loss is a common chronic condition among aging adults that can have meaningful impacts on individuals and society (Cruickshanks et al., 1998a; Agrawal et al., 2008; Nash et al., 2011; McDaid et al., 2021; Dillard et al., 2025). Age-related hearing loss is associated with various negative consequences related to communication and quality of life, as well as poorer general health and cognition (Dalton et al., 2003; Gopinath et al., 2009a; Ciorba et al., 2012; Dillard et al., 2023). Given that the population is aging (US Census, 2025), the caseload of hearing loss is projected to rise dramatically (Goman et al., 2017), which could lead to challenges for health care systems to provide hearing-related services. Therefore, it is critical to identify modifiable risk factors for age-related hearing loss, which could inform preventive interventions to delay and/or reduce its burden.

Several epidemiological studies have identified smoking as a possible modifiable risk factor for age-related hearing loss. Most of this evidence comes from cross-sectional studies (Cruickshanks et al., 1998b; Agrawal et al., 2008, 2009; Zhan et al., 2011; Helzner et al., 2011; Dawes et al., 2014; Hong et al., 2015; Baiduc et al., 2022; Morales et al., 2022), although some longitudinal studies report associations between smoking and the incidence or progression of hearing loss (Nakanishi et al., 2000; Shargorodsky et al., 2010; Cruickshanks et al., 2015; Hu et al., 2019; Lin et al., 2020; Lee et al., 2021; Mick et al., 2023; Uraguchi et al., 2025). However, results remain equivocal, with several cross-sectional and longitudinal studies reporting no association of smoking with hearing loss (Gates et al., 1993; Brant et al., 1996; Karlsmose et al., 2000; Gopinath et al., 2010; Lin et al., 2011; Rigters et al., 2018). Moreover, in some studies, associations of smoking with hearing loss do not remain after adjustment for demographic, hearing-related and health-related confounders, such as noise exposure and/or cardiovascular risk factors (Nash et al., 2011; Dillard et al., 2025). These findings suggest that associations of smoking with hearing could be attributed to other factors, rather than to smoking itself. In addition, it could mean that the observed associations are attributable to downstream impacts of smoking, including poorer cardiovascular health, which could be a stronger predictor of hearing loss (Dillard et al., 2022). Previous epidemiological research has also attempted to evaluate whether smoking cessation could reduce the possible impacts of smoking on hearing. Several longitudinal studies suggest that the risk of incident hearing loss is lower among individuals with a longer time since smoking cessation (Cruickshanks et al., 2015; Hu et al., 2019; Lin et al., 2020).

Across these epidemiological studies, hearing is measured by self-report, speech-in-noise testing, or by audiometric hearing. Audiometric hearing is often defined in terms of a pure-tone average that captures thresholds most important for speech understanding (Brant et al., 1996; Cruickshanks et al., 2015; Mick et al., 2023), and less commonly, by separate pure-tone thresholds at a few selected frequencies (Nakanishi et al., 2000; Sumit et al., 2015; Uraguchi et al., 2025). Although pure-tone average is often regarded as the gold standard measure of hearing, particularly to detect associations with possible risk factors, it lacks the granularity to understand how hearing changes across the entire frequency range (Dillard et al., 2024a). Evaluating associations of smoking with hearing across the entire frequency range could clarify whether the natural history of hearing loss is influenced by smoking. Relatedly, it could determine how smoking may influence the configuration (in addition to the severity) of hearing loss, which could, in turn, improve understanding on the underlying mechanisms and treatment benefits.

Therefore, the purpose of this longitudinal community-based cohort study was to determine the association of smoking with the rate of age-related hearing decline across the frequency range.

## 2. Methods

### 2.1. Study sample

The Medical University of South Carolina (MUSC) Longitudinal Cohort Study of Age-related Hearing Loss is an ongoing (1988-current) community-based cohort study based in Charleston, SC, USA. Participants are continuously enrolled into the cohort and must be aged 18 years or older, and in good general health with no evidence of conductive hearing loss or active otologic or neurologic disease. Previous publications describe study methods and key research findings in detail (Matthews et al., 1997; Dubno et al., 2008; Simpson et al., 2019; Dillard et al., 2024a, 2025a, 2025b).

At the time of analyses, there were 1788 participants with baseline data. To be included in this study, participants were required to have audiometric data from at least two time points and have complete data to ascertain smoking history (described later). All participants provided written informed consent and all protocols were approved by the Institutional Review Board at MUSC (approval ID: Pro00143394).

### 2.2. Audiometric testing

Pure-tone audiometric thresholds at frequencies 0.25, 0.5, 1.0, 2.0, 3.0, 4.0, 6.0, and 8.0 kHz were measured with a clinical audiometer equipped with TDH-39 headphones (Telephonics Corporation, Farmingdale, NY, USA) in a sound-treated booth. Audiological equipment is calibrated to the appropriate American National Standards Institute (ANSI) standards annually (ANSI, 2018). All testing was conducted by ASHA-certified audiologists or Au.D. externs under the supervision of certified audiologists. Thresholds were measured in 5-dB steps, following American Speech-Language Hearing Association standards (ASHA, 2005).

Threshold values from the right and left ears were averaged, following descriptive analyses that showed similar mean threshold values in both ears (Dillard et al., 2024a, 2025c). A pure-tone average (PTA) was calculated from averaged right and left ear threshold values at frequencies 0.5, 1.0, 2.0 and 4.0 kHz. The outcome measures for this study were individual audiometric thresholds at 0.25 to 8.0 kHz and PTA.

### 2.3. Smoking history

Participants reported their smoking history as never smoker, past smoker, or current smoker, the number of cigarettes, cigars, or tobacco pipes smoked per day, and the number of years smoked. Smoking pack years were calculated as the number of cigarettes or cigars smoked per day divided by 20 (one pack) multiplied by the number of years smoked. Tobacco pipe use was converted into pack equivalents and pack years were calculated the same way. Smoking pack years were categorized as 0, >0 to 5, >5 to 15, and >15 pack years. Among past smokers, we calculated the time, in years, since smoking cessation as the year of participants’ final examination minus the year they reported stopping smoking. Time since smoking cessation was categorized in 5-year increments, as follows ≤5, >5 to 10, >15 to 20, and >20 years.

### 2.4. Covariates

Participants reported their age, sex assigned at birth (male/female), and race according to US Census Bureau classifications (American Indian or Alaska Native, Asian, Black or African American, Native Hawaiian or Other Pacific Islander, White, or Other race) (US Census, 2022). Race was categorized as White, Black, or Other race, which was the aggregate of all other races reported, to retain appropriate sample sizes for reporting and analysis.

### 2.5. Statistical methods

All statistical analyses were conducted in SAS version 9.4 software (Cary, NC). We used descriptive statistics to characterize the study sample. We used chi-square tests for categorical variables and one-way analysis of variance for continuous variables to determine differences in sample characteristics (1) between participants who were included and excluded from this study, and (2) among participants across smoking pack years categories. We present age-adjusted mean audiometric thresholds for each smoking pack years group, generated from least squares means estimates from generalized linear regression models.

We used linear mixed regression models, adjusted for baseline age, sex, and race, to estimate the effect of age (per +1 year) on the progression of hearing loss at each frequency, separately, and PTA, for each smoking pack years category. In preliminary analyses (focused on PTA) to inform the selection of final models, we evaluated whether the estimates were changed by including socioeconomic position and/or noise exposure history in the models (Dillard et al., 2024a, 2024b, 2025a, 2025b). Inclusion of these covariates did not meaningfully change the magnitude or significance of the estimates generated from models adjusted only for baseline age, sex, and race. Therefore, we did not include additional covariates (other than baseline age, sex, and race) in our final models, also because doing so would have substantially reduced the sample size due to missing covariate data.

Age (at each examination) was treated as the repeated time measure. Therefore, rate is interpreted as the rate of change per one year in age. Linear mixed models are ideal for repeated measures data with varying lengths of follow-up time, and these models manage fixed (population-level) parameters, which are shared by the study population, and random (participant-specific) parameters, which can vary across individuals (Pinhiero et al., 2000). To determine the association of smoking pack years with the rate of threshold change, we included interaction terms of age, the longitudinal time variable, and smoking pack years (referent group = 0 pack years) in the regression models. In addition, to determine whether the association between smoking pack years and the rate of PTA change varied by baseline age group (categorized as ≤65 years, >65 to ≤75 years, and >75 years), we included a three-way interaction term between these three variables in a separate model. A sensitivity analysis evaluated the association of smoking pack years with the rate of PTA change after excluding the 22 participants who smoked cigars or tobacco pipes, rather than cigarettes. Results from all models are presented as regression coefficients with corresponding 95 % confidence intervals (CI).

Lastly, we determined the association of time since smoking cessation, among past smokers, with the rate of PTA change, using methods similar to those detailed above. These models were first adjusted for baseline age, sex, and race, then additionally adjusted for smoking pack years.

## 3. Results

Of the 1778 participants with baseline data, 1032 met the eligibility criteria and were included in this study. Participants included in this study (versus excluded) were more likely to be older (*p* < 0.01), female (*p* < 0.01), and White race (*p* < 0.01) but did not differ by PTA (*p* = 0.91).

Table 1 shows the baseline characteristics of the 1032 participants, overall and stratified by pack years categories. Participants’ mean baseline age was 63.3 (SD 14.1) years, 60.4 % were female, and 22.5 % were racial Minority (21.7 % of the sample was Black race and 0.8 % was Other race). The mean length of follow-up time was 5.4 (SD 6.0) years. Some participant characteristics differed across smoking pack years categories, including age (*p* < 0.01), sex (*p* < 0.01), race (*p* < 0.01), and number of audiograms (*p* < 0.01). Participants with >15 smoking pack years were slightly older than those in the other categories. Participants who were male and White race were more likely to have more smoking pack years. In addition, years since smoking differed across smoking pack years categories (>0 to 5, >5 to 15, >15 years only; *p* < 0.01), and participants with >0 to 5 pack years showed the longest time since smoking (Table 1). Among participants who reported current or past smoking, 96.0 % were cigarette smokers, whereas the remaining 4.0 % (22 participants) reported smoking cigars and/or tobacco pipes.

**Fig. 1.**
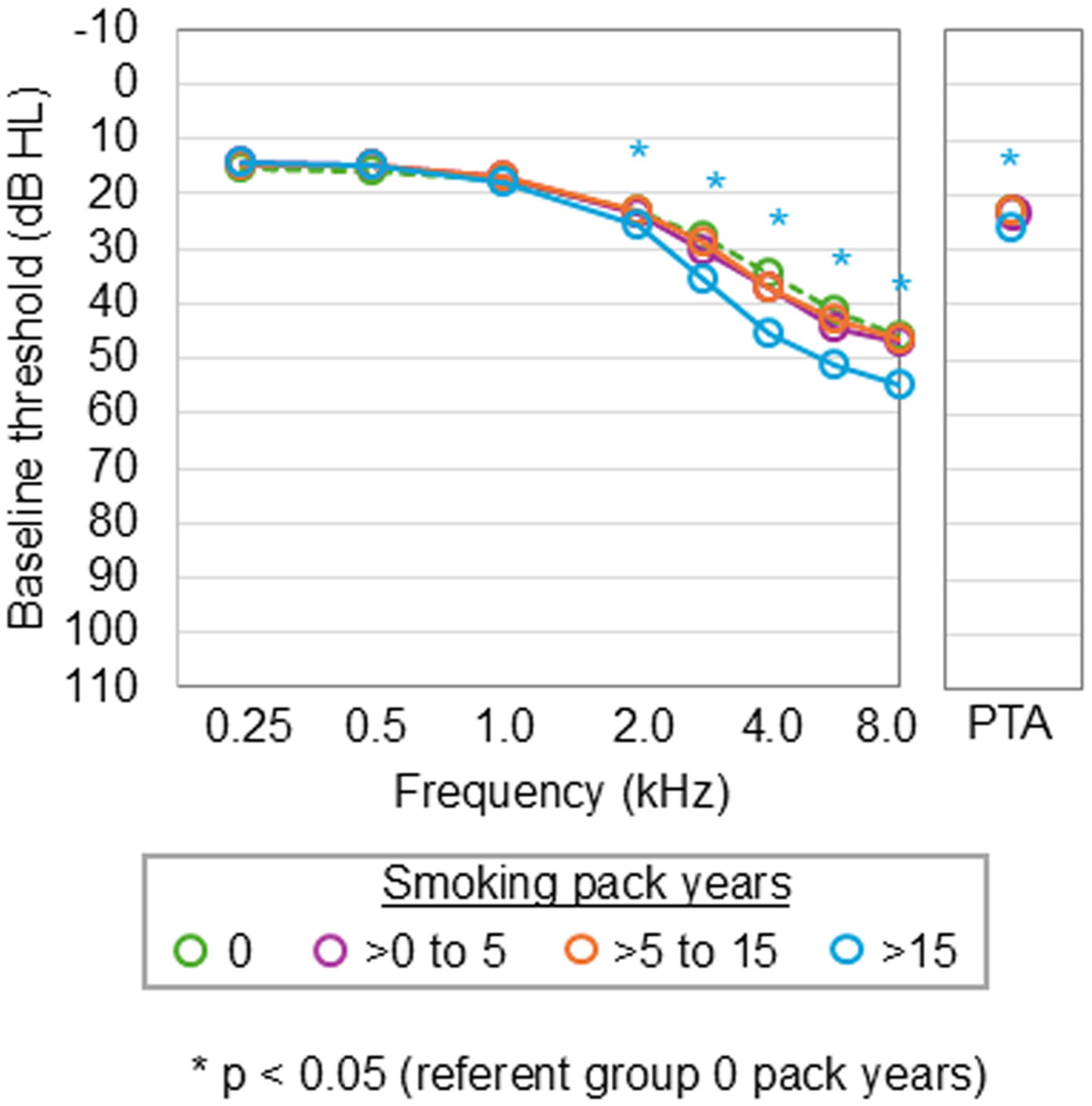
Mean age-adjusted baseline thresholds, by smoking pack years.

Age-adjusted baseline thresholds by smoking pack years are in Fig. 1, with the corresponding estimates in Supplementary Table 1. Baseline thresholds at frequencies 0.25, 0.5, and 1.0 kHz were similar across the pack years categories. Compared to non-smokers (0 pack years), participants with >15 pack years had significantly poorer (higher) baseline thresholds at frequencies 2.0 kHz to 8.0 kHz and PTA.

### 3.1. Rate of threshold change by smoking pack years

**Fig. 2.**
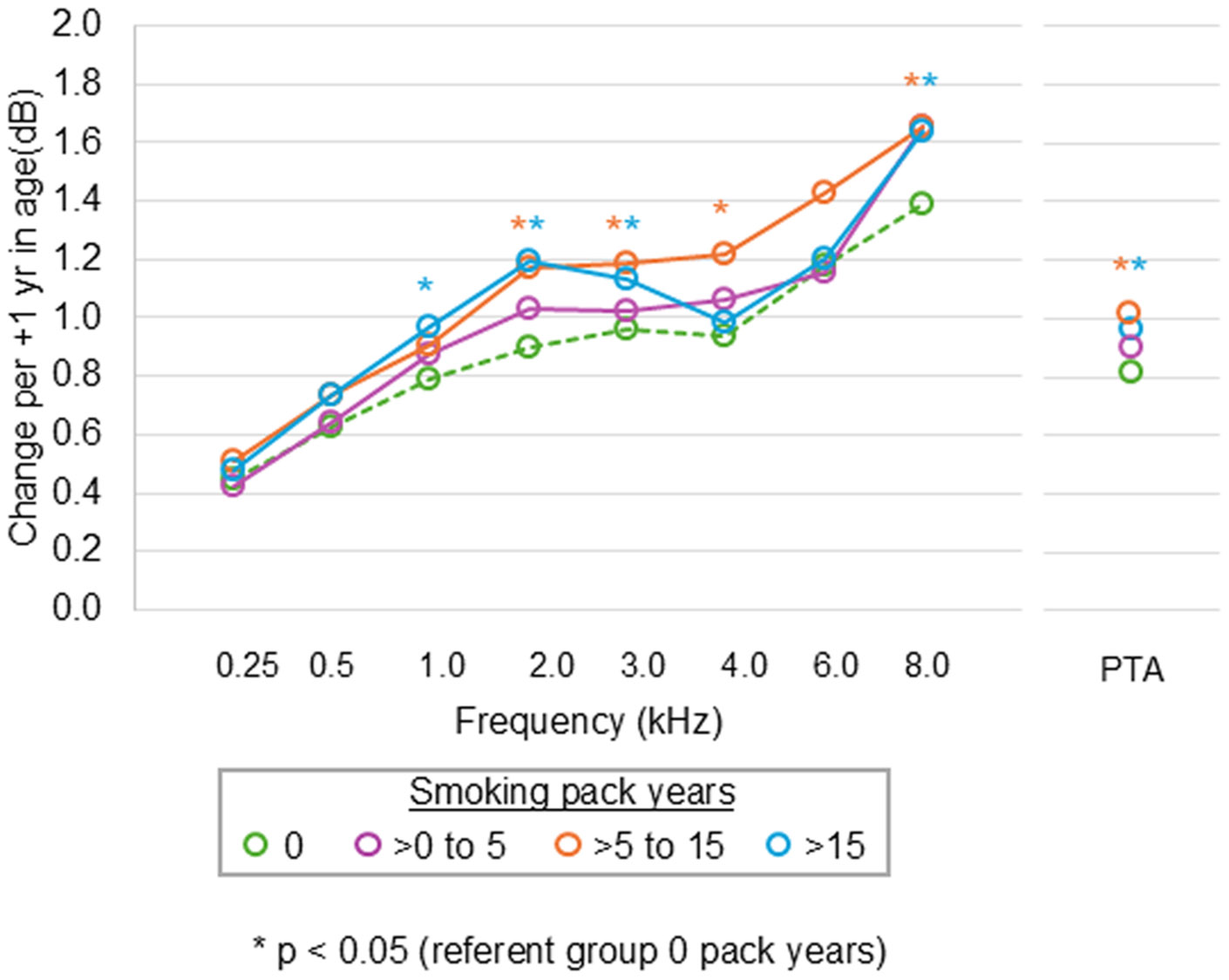
Mean rates of audiometric threshold and PTA change per year by smoking pack years, adjusted for baseline age, sex, and race.

Fig. 2 shows the mean rates of audiometric threshold and PTA change per year, adjusted for baseline age, sex, and race, across pack years categories (corresponding estimates are in Supplementary Table 2). Compared to non-smokers, participants with >5 to 15 pack years show higher rates of change at 2.0, 3.0, 4.0, and 8.0 kHz and PTA. Participants with >15 pack years (versus non-smokers) show higher rates of change at 1.0, 2.0, 3.0, and 8.0 kHz and PTA. Rates of change were similar between non-smokers and participants with >0 to 5 pack years.

There was not evidence that the association between smoking pack years and the rate of PTA change varied by baseline age group, categorized as ≤65, >65 to ≤75, and >75 years (3-way interaction term of smoking pack years, rate of PTA change, and baseline age group: F-statistic: 1.4; *p* = 0.20). In a sensitivity analysis that excluded the 22 smokers who did not smoke cigarettes, results were similar to those presented in Fig. 2 and Supplementary Table 2 (results not shown).

Participants’ age-adjusted thresholds from the baseline and final examinations, across pack years categories, are shown in Supplementary Figure 1. In general, non-smokers show the best hearing thresholds, particularly in the mid to high frequencies, whereas participants with >15 pack years show the poorest thresholds at both the baseline and final examinations.

### 3.2. Time since smoking cessation

Table 2 shows associations of time since smoking cessation, among past smokers compared to never smokers, with the rate of PTA change. Estimates are adjusted for baseline age, sex, and race. The mean rate of PTA change per year was similar for never smokers and those with a time since smoking cessation of ≤5 years (*p* = 0.73). Notably, compared to never smokers, participants with a time since smoking cessation of ≤5 years were, on average, approximately 7 years younger. Compared to never smokers, the mean rate of PTA change was non-significantly higher for participants with a time since smoking cessation of >5 to 10 years (*p* = 0.08), and >15 to 20 years (*p* = 0.19), and was significantly higher for participants with a time since smoking cessation of >10 to 15 years (*p* = 0.04) and >20 years (*p* < 0.01). In general, the rate of PTA change was highest among participants with a time since smoking cessation of >5 to 10 and >10 to 15 years, who also had the highest number of mean pack years. The rate of PTA change was slightly lower among participants with a time since smoking cessation of >15 to 20 and >20 years. Additional adjustment for pack years did not change the magnitude or significance of these associations (Table 2).

## 4. Discussion

Results from this longitudinal community-based cohort study indicate that smoking history, expressed in pack years, is associated with poorer hearing and greater annual declines to hearing in the mid to high frequencies in aging. Findings corroborate smoking as a possible modifiable risk factor for hearing loss that could have lasting impacts on hearing as individuals age.

These results are consistent with several other epidemiological studies showing associations of smoking with the prevalence, incidence, and progression of hearing loss (Cruickshanks et al., 1998b, 2015; Nakanishi et al., 2000; Agrawal et al., 2009; Shargrodsky et al., 2010; Zhan et al., 2011; Helzer et al., 2011; Dawes et al., 2014; Hong et al., 2015; Hu et al., 2019; Lin et al., 2020; Lee et al., 2021; Baiduc et al., 2022; Morales et al., 2022; Mick et al., 2023; Uraguchi et al., 2025). This study is novel because it comprehensively evaluates associations of smoking with hearing across the entire frequency range (0.25 to 8.0 kHz), as well as PTA. Evaluating these associations across the frequency range clarified that cross-sectional and longitudinal associations were present in the mid to high, but not the low frequencies. This suggests that, similar to the effect of other risk factors for hearing loss, such as noise exposure, smoking may first impact hearing in the high frequencies. Moreover, changes to hearing across frequencies followed a similar pattern to those observed in age-related hearing loss (Eckert et al., 2021). In addition, several cross-sectional studies suggest that passive smoking/secondhand smokeis associated with hearing loss in non-smokers and former smokers (Cruickshanks et al., 1998; Fabry et al., 2010; Dawes et al., 2014). The detrimental effects of risk factors for hearing loss, including active and/or passive smoking, could compound over the life course, resulting in poorer high-frequency hearing loss in aging.

Consistent with some previous research, poorer baseline hearing and hearing declines were most pronounced among heavier smokers, and there was no association of smoking with hearing loss or decline among participants with <5 pack years (Cruickshanks et al., 1998b; Nakanishi et al., 2000; Agrawal et al., 2009; Dawes et al., 2014; Hu et al., 2019). In general, these findings suggest a possible dose-response relationship between smoking and hearing loss; that is, higher levels of exposure to smoking could lead to more severe hearing loss. However, for some frequencies, participants with >5 to 15 pack years showed the highest rates of hearing decline (higher than participants with >15 pack years). There are several possible explanations for this finding. First, the baseline thresholds at the higher frequencies were already elevated among participants with >15 pack years; therefore, consistent with patterns observed in aging and after exposure to other risk factors such as noise, hearing loss may be progressing to the lower frequencies (including 1.0 kHz). Similar trends have been reported across other studies in this cohort (Dillard et al., 2024a, 2025c). Second, participants with >5 to 15 pack years had the highest proportion of current smokers (31.1 %; compared to 13.5 % among participants with >15 pack years; see Table 1). Current smokers may be the most susceptible to damage to hearing given their consistent exposure to cigarette smoke and nicotine.

Previous studies have evaluated associations of smoking, defined by both smoking pack years and/or smoking status (current, past, never), with hearing (Sharorodsky et al., 2010; Cruickshanks et al., 2015; Hu et al., 2019; Lin et al., 2020). In this study, we expressed smoking history in pack years for several reasons. First, using pack years captures life-time exposure to smoking, the effects of which could compound over the life course. Second, defining smoking with pack years ensured that the age distribution was relatively even across comparison groups, although participants with >15 pack years were slightly older (Table 1). This reduced the risk of confounding by age, which is important because age is strongly associated with hearing thresholds (Cruickshanks et al., 1998a; Agrawal et al., 2008; Nash et al., 2011; Eckert et al., 2021; Dillard et al., 2025a). In this sample, there were substantial differences in the mean ages among past (66.7 [SD 10.6] years), current (54.8 [SD 12.9] years) and never (62.1 [SD 16.0] years) smokers. While expressing cigarette smoking in pack years was appropriate for these analyses, using this construct may have limited our ability to evaluate the acute effects of smoking on hearing, which is likely best captured by smoking status at the time of the examination or measured cotinine levels (a biomarker of nicotine exposure) (Nondahl et al., 2004; Lee et al., 2021). Indeed, some studies have reported associations of smoking status (never, current, past), but not pack years, with hearing loss (Cruickshanks et al., 2015). Other studies have reported associations of both smoking status and pack years with hearing loss (Lin et al., 2020).

In this study, we attempted to evaluate the association of time since smoking cessation with the rate of hearing decline. In general, results show a trend that participants who stopped smoking >15 to 20 or >20 years prior had slightly lower rates of decline than those who stopped smoking >5 to 10 or >10 to 15 years prior. However, results are difficult to interpret given differences in the mean pack years among groups with varying times since smoking cessation. Although these trends were consistent after additional adjustment for pack years, it is possible that the substantial differences in pack years among these groups could influence the results. Taken together, results from this study do not provide clear evidence to support that smoking cessation slows the rate of hearing decline. Only a few longitudinal studies evaluate the relationship between the time since smoking cessation and incident hearing loss or hearing decline (Cruickshanks et al., 2015; Hu et al., 2019; Lin et al., 2020), all of which showed that individuals with a longer time since smoking cessation showed a lower risk of hearing loss (Cruickshanks et al., 2015; Hu et al., 2019; Lin et al., 2020). Smoking cessation could reduce the risk of cardiovascular disease, stroke, and cancer, all of which have been associated with hearing loss (Gates et al., 1993; Gopinath et al., 2009b; Mick et al., 2023; Dillard et al., 2025d). Conversely, some cross-sectional studies did not report an association between smoking cessation and hearing thresholds (Fabry et al., 2010; Morales et al., 2022). Some studies in other sensory-related areas, including olfaction and vision, have reported associations of smoking cessation and reduced risk of sensory disorders, but only after long periods (at least 15 to 20 years) since smoking (Lindblad et al., 2005; Siegel et al., 2019). More research is needed to understand the possible impacts of smoking cessation on sensory changes, including hearing, in aging.

As mentioned earlier, while there is compelling evidence from longitudinal studies showing associations of smoking with hearing loss incidence and progression (Cruickshanks et al., 2015; Hu et al., 2019; Lin et al., 2020), several studies do not report an association (Brant et al., 1996; Karlsmose et al., 2000; Gopinath et al., 2010; Linssen et al., 2014; Rigters et al., 2018). Reasons for the inconsistencies in findings across studies could be explained by differences in study designs, including varying definitions of smoking and hearing and varying lengths of follow-up times. In addition, inconsistencies could be explained by varying distributions of demographic characteristics of the samples, including in terms of age, sex, and race, all of which are associated with hearing loss (Cruickshanks et al., 1998a; Agrawal et al., 2008; Nash et al., 2011; Helzner et al., 2011; Dillard et al., 2025a). Lastly, there could be systematic differences in smoking habits across cohorts, which is supported by research suggesting regional differences, both across and within countries, in the prevalence of smoking (Doescher et al., 2006; Islami et al., 2015; Doogan et al., 2017).

There are several hypothesized mechanisms that could explain the observed associations between smoking and hearing. Cigarette smoke and nicotine may be both ototoxic and neurotoxic (Durante et al., 2013; Abdel-Hafez et al., 2014; Paquette et al., 2018), which could explain associations of smoking with sensory disorders, including hearing loss, and poorer cognitive function (Lindblad et al., 2005; Durazzo et al., 2014; Siegel et al., 2019). Results from animal studies suggest that these substances could lead to direct damage to the cochlear spiral ganglion and outer hair cells, through mechanisms including increased oxidative stress, cell injury or death, and/or cochlear hypoxia (Pryor et al., 1993; Garbin et al., 2009; Durante et al., 2013; Abdel-Hafez et al., 2014; Lisowska et al., 2017; Paquette et al., 2018). In addition, smoking is a risk factor for inflammatory conditions, including atherosclerosis, and poor cardiovascular and metabolic health, all of which have been associated with increased hearing loss (Nash et al., 2011; Cruickshanks et al., 2015; Fischer et al., 2015; Schubert et al., 2019; Mick et al., 2023). Lastly, some studies suggest that smoking may increase susceptibility to noise-induced hearing loss, although others suggest that the effects of the two exposures are additive (Mizoue et al., 2003; Li et al., 2020).

In general, results suggest that smoking may be a modifiable risk factor for hearing loss. Although the overall prevalence of smoking has declined among adults in the United States, prevalence estimates of smoking remain stable among older adults aged 65 years and older, the group at highest risk for hearing loss (Meza et al., 2023). Although the prevalence of smoking is decreasing in most of the world, smoking cessation remains a domestic and global health priority given its wide-ranging negative impacts on health (West, 2017; WHO, 2018). As smoking trends continue to evolve, particularly among young people, it will be necessary to evaluate whether other / related substances, such as e-cigarettes (vaping), or cannabis use, may be modifiable risk factors for hearing loss (Baiduc et al., 2022; Bener et al., 2024; Anand et al., 2024).

Strengths of this study include its large and multi-racial sample of the general population and its longitudinal design spanning up to 35 years with annual measurements of audiometric thresholds. This cohort is similar to other epidemiological cohort studies of hearing in terms of age and audiometric hearing, which could enhance the generalizability of findings (Cruickshanks et al., 1998a; Gates et al., 1990; Dubno et al., 2008). The prevalence of current smokers in this study (9.8 %) is similar to the national prevalence (Meza et al., 2023). However, some limitations exist. First, this study was conducted in a relatively small geographic region, which could limit generalizability of findings. Second, as in all observational studies, there is potential for residual confounding and therefore, differences across smoking pack years groups could be partially explained by other, unmeasured, confounders (Fewell et al., 2007). In addition, although all models were adjusted for demographic factors, there were differences in participant characteristics across pack years categories (Table 1; e.g., in terms of age, sex, and race), which likely reflect population-level trends in smoking histories but could influence results. There is potential for survivorship bias and/or recall or social desirability bias, as smoking was self-reported. However, despite variability across studies, the sensitivity and specificity of self-reported smoking, compared to measured biomarkers, are often high (Patrick et al., 1994; Gorber et al., 2009). Although our models adjusted only for demographic factors (age, sex, and race), results from our preliminary models did not suggest that adjusting for other possible confounders, including socioeconomic position and/or noise exposure, resulted in meaningful changes to estimates.

## 5. Conclusions

In this longitudinal community-based cohort study of the general population, smoking history was associated with poorer hearing and greater annual declines to hearing in aging. Smoking could have lasting impacts on hearing and may be a modifiable risk factor for age-related hearing loss.

## Supplementary Material

1

Supplementary material associated with this article can be found, in the online version, at doi:10.1016/j.heares.2025.109486.

Supplemental file legend

Supplementary files include (1) mean age-adjusted baseline thresholds, (2) mean rates of audiometric and PTA change, and (3) mean age-adjusted baseline and audiometric thresholds.

## Figures and Tables

**Table 1:** Baseline characteristics for the 1032 participants in the MUSC Longitudinal Cohort Study of Age-related Hearing Loss. Results are presented as mean (SD) or n (%).

	Entire sample	Smoking pack years				F-statistic or χ^2^, p-value
		0 years	>0 to 5 years	>5 to 15 years	>15 years	
Number	1032	486	206	132	208	
Age (years)	63.3 (14.1)	62.1 (16.0)	63.0 (14.2)	61.8 (11.0)	67.7 (9.2)	8.6, *p* < 0.01
Sex						72.3, *p* < 0.01
Female	623 (60.4 %)	348 (71.6 %)	126 (61.2 %)	70 (53.0 %)	79 (38.0 %)	
Male	409 (39.6 %)	138 (28.4 %)	80 (38.8 %)	62 (47.0 %)	129 (62.0 %)	
Race						30.8, *p* < 0.01
White	800 (77.5 %)	370 (76.1 %)	161 (78.2 %)	84 (63.6 %)	185 (88.9 %)	
Black	224 (21.7 %)	109 (22.4 %)	44 (21.4 %)	48 (36.4 %)	23 (11.1 %)	
Other^†^	8 (0.8 %)	‡	‡	‡	‡	
Time in study (years)	5.4 (6.0)	5.1 (6.1)	5.5 (5.8)	5.2 (6.1)	6.3 (5.8)	2.0, *p* = 0.11
n of audiograms						30.4, *p* < 0.01
2 to 5	453 (43.9 %)	245 (50.4 %)	85 (41.3 %)	58 (43.9 %)	65 (31.3 %)	
6 to 10	240 (23.3 %)	98 (20.2 %)	44 (21.4 %)	36 (27.3 %)	62 (29.8 %)	
11 to 15	145 (14.1 %)	58 (11.9 %)	39 (18.9 %)	12 (9.1 %)	36 (17.3 %)	
16 to 20	103 (10.0 %)	44 (9.1 %)	21 (10.2 %)	13 (9.9 %)	25 (12.0 %)	
21+	91 (8.8 %)	41 (8.4 %)	17 (8.3 %)	13 (9.9 %)	20 (9.6 %)	
Smoking history						18.5, *p* < 0.01^§^
Never	486 (47.1 %)	486 (47.1 %)	0 (0 %)	0 (0 %)	0 (0 %)	
Past	445 (43.1 %)	0 (0 %)	174 (84.5 %)	91 (68.9 %)	180 (86.5 %)	
Current	101 (9.8 %)	0 (0 %)	32 (15.5 %)	41 (31.1 %)	28 (13.5 %)	
Years since smoking	28.4 (15.2)	–	34.0 (16.0)	25.3 (13.7)	24.4 (12.2)	21.3, *p* < 0.01^§^

† Other race categories includes American Indian or Alaska Native, Asian, Native Hawaiian or Other Pacific Islander, or Other race.

‡ Not reported due to low sample sizes.

§ Participants with 0 pack years were excluded from these analyses.

**Table 2:** Mean rates of PTA change per year (dB) by time since smoking cessation, compared to never smokers.

Time since smoking cessation (year)			Mean pack years (SD)	Adjusted for baseline age, sex, and race		Adjusted for baseline age, sex, race, and pack years	
	n	Mean age (SD), years		B (95 % CI)	p-value	B (95 % CI)	p-value
Never smoker	486	62.1 (16.0)	0	0.82 (0.75, 0.88)	1.00 (Referent)	0.82 (0.75, 0.88)	1.00 (Referent)
≤5 years	46	55.5 (18.6)	22.2 (30.4)	0.78 (0.56, 1.01)	0.73	0.79 (0.56, 1.01)	0.69
>5 to 10 years	31	62.9 (12.4)	32.9 (36.5)	1.13 (0.77, 1.50)	0.08	1.12 (0.74, 1.49)	0.08
>10 to 15 years	30	64.2 (10.8)	34.9 (36.2)	1.06 (0.84, 1.28)	0.04	1.05 (0.83, 1.28)	0.04
>15 to 20 years	52	65.4 (8.6)	24.7 (27.0)	0.92 (0.72, 1.12)	0.19	0.92 (0.72, 1.22)	0.21
>20 years	286	69.4 (7.0)	15.1 (20.1)	0.96 (0.88, 1.03)	<0.01	0.96 (0.88, 1.03)	<0.01

Note. PTA is defined as the average of thresholds at 0.5, 1.0, 2.0 and 4.0 kHz, averaged bilaterally. p-value is from the interaction term of years since smoking cessation x age (the longitudinal time variable), compared to the referent group of never smokers.

## Data Availability

Deidentified participant data are available upon reasonable request to the corresponding author under a data use agreement and institutional approvals according to guidelines of the Medical University of South Carolina.
