# Supplementary material for: Smoking history and the rate of hearing decline in aging: Results from a longitudinal cohort study
Source: Hear Res. Author manuscript; Available in PMC 2026 Jun 26. (PMC13308747; doi:10.1016/j.heares.2025.109486)
Supplement: 1 [file NIHMS2187137-supplement-1.docx]

Supplementary Table 1: Mean age-adjusted baseline thresholds (dB HL), with 95% confidence intervals, by smoking pack years. Values correspond to those plotted in Figure 1.

|  | Smoking pack years | | | |
| --- | --- | --- | --- | --- |
|  | 0 years | >0 to 5 years | >5 to 15 years | >15 years |
| Frequency |  |  |  |  |
| 0.25 kHz | 15.3 (14.4, 16.2) | 14.5 (13.2, 15.9) | 14.7 (13.0, 16.4) | 14.2 (12.9, 15.6) |
| 0.50 kHz | 15.9 (14.9, 16.8) | 14.7 (13.2, 16.2) | 14.9 (13.1, 16.8) | 14.7 (13.2, 16.2) |
| 1.0 kHz | 17.4 (16.2, 18.5) | 16.9 (15.2, 18.6) | 17.0 (14.8, 19.1) | 17.9 (16.2, 19.6) |
| 2.0 kHz | 23.4 (22.0, 24.7) | 23.5 (21.4, 25.7) | 23.1 (20.4, 25.8) | 25.9 (23.8, 28.1) |
| 3.0 kHz | 28.0 (26.4, 29.6) | 30.3 (27.8, 32.7) | 29.1 (26.1, 32.2) | 35.6 (33.2, 38.1) |
| 4.0 kHz | 34.4 (32.7, 36.2) | 37.2 (34.6, 39.9) | 37.4 (34.0, 40.7) | 45.5 (42.8, 48.1) |
| 6.0 kHz | 41.5 (39.7, 43.3) | 44.7 (41.9, 47.4) | 43.0 (39.6, 46.4) | 51.4 (48.7, 54.2) |
| 8.0 kHz | 45.9 (44.1, 47.7) | 47.3 (44.5, 50.1) | 46.6 (43.1, 50.1) | 55.0 (52.2, 57.9) |
| PTA (dB HL) | 22.7 (21.7, 23.8) | 23.1 (21.4, 24.7) | 23.1 (21.0, 25.2) | 26.0 (24.3, 27.7) |

Note. PTA is defined as the average of thresholds at 0.5, 1.0, 2.0 and 4.0 kHz, averaged bilaterally.

Supplementary Table 2: Mean rates of audiometric threshold and PTA change per year (dB) by smoking pack years, presented as regression coefficients with 95% confidence intervals. Values correspond to those plotted in Figure 2.

|  | Smoking pack years | | | |
| --- | --- | --- | --- | --- |
|  | 0 years | >0 to 5 years | >5 to 15 years | >15 years |
| Frequency |  |  |  |  |
| 0.25 kHz | 0.45  (0.37, 0.53) | 0.42  (0.32, 0.53) | 0.51  (0.36, 0.66) | 0.48  (0.37, 0.58) |
| 0.50 kHz | 0.63  (0.55, 0.70) | 0.64  (0.52, 0.76) | 0.73  (0.58, 0.89) | 0.73  (0.63, 0.84) |
| 1.0 kHz | 0.79  (0.71, 0.87) | 0.88  (0.76, 1.00) | 0.90  (0.75, 1.06) | 0.97  (0.84, 1.11) |
| 2.0 kHz | 0.90  (0.81, 0.99) | 1.03  (0.88, 1.18) | 1.17  (0.97, 1.37) | 1.19  (1.06, 1.32) |
| 3.0 kHz | 0.96  (0.88, 1.05) | 1.03  (0.89, 1.17) | 1.18  (1.00, 1.37) | 1.13  (1.00, 1.26) |
| 4.0 kHz | 0.94  (0.85, 1.03) | 1.06  (0.93, 1.18) | 1.22  (1.04, 1.40) | 0.99  (0.86, 1.12) |
| 6.0 kHz | 1.18  (1.07, 1.29) | 1.15  (0.98, 1.33) | 1.43  (1.19, 1.66) | 1.20  (1.04, 1.36) |
| 8.0 kHz | 1.39  (1.28, 1.51) | 1.66  (1.47, 1.84) | 1.65  (1.45, 1.86) | 1.64  (1.48, 1.80) |
| PTA (dB HL) | 0.82  (0.75, 0.88) | 0.91  (0.81, 1.00) | 1.02  (0.87, 1.16) | 0.97  (0.87, 1.06) |

Note. Estimates are adjusted for baseline age, sex and race. PTA is defined as the average of thresholds at 0.5, 1.0, 2.0 and 4.0 kHz, averaged bilaterally.

Supplementary Figure 1: Baseline and final audiometric thresholds by smoking pack years category, adjusted for age.


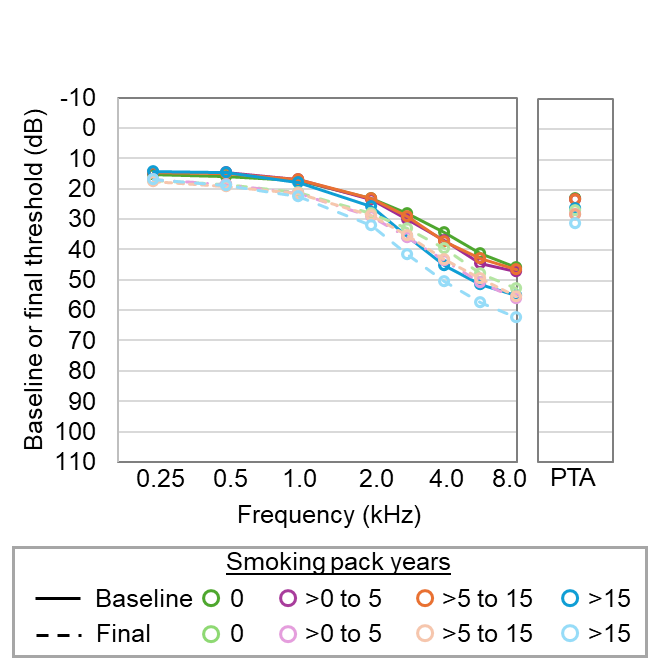


Note. PTA is defined as the average of thresholds at 0.5, 1.0, 2.0 and 4.0 kHz, averaged bilaterally.
